# Perception, practice, and barriers toward research among pediatric undergraduates: a cross-sectional questionnaire-based survey

**DOI:** 10.1186/s12909-024-05361-x

**Published:** 2024-04-03

**Authors:** Canyang Zhan, Yuanyuan Zhang

**Affiliations:** 1 Department of Neonatology, Children’s Hospital, Zhejiang University School of Medicine, National Clinical Research Center for Child Health, Hangzhou, China; 2 Department of Pulmonology, Children’s Hospital, Zhejiang University School of Medicine, National Clinical Research Center for Child Health, Hangzhou, China

**Keywords:** Pediatrics, Practices, Barrier, Undergraduate research, Medical research

## Abstract

**Background:**

Scientific research activities are crucial for the development of clinician-scientists. However, few people pay attention to the current situation of medical research in pediatric medical students in China. This study aims to assess the perceptions, practices and barriers toward medical research of pediatric undergraduates.

**Methods:**

This cross-sectional study was conducted among third-year, fourth-year and fifth-year pediatric students from Zhejiang University School of Medicine in China via an anonymous online questionnaire. The questionnaires were also received from fifth-year students majoring in other medicine programs [clinical medicine (“5 + 3”) and clinical medicine (5-year)].

**Results:**

The response rate of pediatric undergraduates was 88.3% (68/77). The total sample of students enrolled in the study was 124, including 36 students majoring in clinical medicine (“5 + 3”) and 20 students majoring in clinical medicine (5-year). Most students from pediatrics (“5 + 3”) recognized that research was important. Practices in scientific research activities are not satisfactory. A total of 51.5%, 35.3% and 36.8% of the pediatric students participated in research training, research projects and scientific article writing, respectively. Only 4.4% of the pediatric students contributed to publishing a scientific article, and 14.7% had attended medical congresses. None of them had given a presentation at a congress. When compared with fifth-year students in the other medicine program, the frequency of practices toward research projects and training was lower in the pediatric fifth-year students. Lack of time, lack of guidance and lack of training were perceived as the main barriers to scientific work. Limited English was another obvious barrier for pediatric undergraduates. Pediatric undergraduates preferred to participate in clinical research (80.9%) rather than basic research.

**Conclusions:**

Although pediatric undergraduates recognized the importance of medical research, interest and practices in research still require improvement. Lack of time, lack of guidance, lack of training and limited English were the common barriers to scientific work. Therefore, research training and English improvement were recommended for pediatric undergraduates.

## Background

Medical education includes the learning of basic clinical medical knowledge and the cultivation of scientific research abilities. Scientific research, an essential part of medical education, is increasingly important, as it can greatly improve medical care [1, 2]. Scientific research activities are crucial for the development of clinician-scientists, who have key roles in clinical research and translational medicine. Therefore, medical education is increasingly emphasizing the cultivation of scientific research abilities. Strengthening scientific research training helps students to develop independent critical thinking, improve the ability of observation, and foster the problem-solving skills. It is suggested that developing undergraduate research benefits the students, the faculty mentors, the university or institution, and eventually society [2, 3]. As a result, there is a growing trend to integrate scientific research training into undergraduate medical education. Early exposure to scientific research was recommended in undergraduate medical students [4, 5]. In fact, an international questionnaire study showed that among 1625 responses collected from 38 countries, less than half (42.7%) agree/strongly agree that their medical schools provided “sufficient training in medical research” [6]. The training or practices about medical research in undergraduates is not universal. In China, few people pay attention to the current situation of medical research in undergraduates, especially for pediatric medical students.

Due to changes in China’s birth policy (two-child policy in 2016 and the three-child policy in 2021), child health needs are increasing [7]. The shortage of pediatricians is alarming in China. Therefore, numerous policies have been implemented to meet the challenges of the shortage of pediatricians, including reinstating pediatrics as an independent discipline in medical school enrollment and increasing the enrollment of pediatrics. The number of pediatricians has increased year by year. The number of pediatricians in China increased from 118,500 in 2015 (0.52 pediatricians per 1000 children under the age of 14) to 206,000 in 2021 (0.78 pediatricians per 1000 children under the age of 14). With the increase in pediatric enrollment, pediatric medical education is facing new challenges. It is urgent to study the current situation of cultivation of pediatric medical students, one of which is the scientific research abilities [8, 9]. However, as the particular background of pediatrics, very little is known about the perception, practice and barriers toward medical research in pediatric undergraduates. The purpose of this study was to address the gap by assessing the practices, perceptions and barriers toward medical research of pediatric undergraduates at Zhejiang University. The results can help to improve the mode of cultivating scientific research abilities among pediatric medical students.

## Methods

The study was conducted from March to April 2023. The study was approved by the Ethics Review Committee of the Children’s Hospital of Zhejiang University School of Medicine and was undertaken according to the Helsinki declaration. Participants provided written informed consent upon applying to participate in the study.

### Study design and setting

This is a cross-sectional study conducted via an online questionnaire and the questionnaire was done simultaneously in all students. The study aimed to investigate the perception, practices and barriers toward research in pediatric undergraduates from Zhejiang University School of Medicine, and to investigate the differences in research among undergraduate students from clinical medicine (“5 + 3” integrated program, pediatrics) [pediatrics (“5 + 3”)], clinical medicine (“5 + 3” integrated program) [clinical medicine (“5 + 3”)] and clinical medicine (5-year).

The clinical medicine of Zhejiang University School of Medicine (ZUSM) includes a 5-year program, a “5 + 3” integrated program, and a 8-year MD. Program. The clinical medicine (5-year) program is the basis of clinical medicine education.Graduates need to complete 3 years of standardized residency training to become doctors. The clinical medicine (“5 + 3”) model combines the 5-year medical undergraduate education, 3-year standardized residency training and postgraduate education. Since 2015, 20 to 30 students who are interested in pediatrics were selected from second-year undergraduate students of clinical medicine (“5 + 3”) to continue studies as pediatrics (“5 + 3”) every year. Since 2019, ZUSM established pediatrics (“5 + 3”) program. 20–30 students have been enrolled independently every year.

### Participants

All of the third-, fourth-, and fifth-year undergraduate students in pediatrics (“5 + 3”) and some of the fifth-year undergraduate students from clinical medicine (“5 + 3”) and clinical medicine (5-year) who expressed an interest in participating in the study were enrolled.

### Data collection

The questionnaire was self-designed after reviewing the literature and consulting senior faculty. For the purpose of testing its clarity and reliability, the questionnaire was pilot tested among 36 undergraduate students. Their feedback was mainly related to the structure of the questionnaire. To address these comments, the questionnaire was modified to reach the final draft, which was distributed to the student sample included in the study. The reliability coefficient was assessed by Cronbach’s alpha, and the validity was evaluated by Kaiser-Meyer-Olkin (KMO).

There are four sections of the questionnaire used in this study:

The first part covered 3 statements (gender, grade and major).

The second part examined the participants’ perceptions of medical research, including 5 statements (importance, enhancement of competitiveness, practising thinking ability, solving clinical problems, and being interesting).

The third part examined practices in medical research, including 6 statements (project, training, write paper, publish paper, attend academic conference and conference communication).

The barriers to medical research were assessed in the last part, including 7 statements.

Perception and barriers toward medical research were evaluated using a five-point Likert scale ranging from 1 to 5 (1 = strongly disagree; 2 = disagree, 3 = uncertain, 4 = agree, 5 = strongly agree).

### Statistical analysis

Categorical data are represented as numbers and frequencies. For ease of reporting and analyzing data, the responses of “agree” and “strongly agree” were grouped and reported as agreements, and “disagree” and “strongly disagree” were grouped as disagreements. The chi-square test was used to test the difference in the frequency of participation in research practices. The student’s perception score based on grades was analyzed using Fisher’s exact test, and attitude between the year of study was analyzed by ANOVA or a nonparametric test (Kruskal-Wallis H test). The statistical analysis was performed using IBM SPSS version 26. *P* < 0.05 was considered significant.

## Results

The reliability coefficient of the questionnaire was assessed by Cronbach’s alpha; it was 0.73 for perception and 0.78 for barriers. KMO was 0.80 for perception (Bartlett’s sphericity test: χ2 = 200.4, *p* < 0.001) and 0.73 for barriers (Bartlett’s sphericity test: χ2 = 278.4, *p* < 0.001), indicating the appropriateness of the factor analysis. The factor analysis was carried out using the principal component analysis with varimax rotation. For perception, one factor explains 58.2% of the variance. For barriers, two-factor solution explains 60.2% of the variance.

The response rate was 79.2% (19/24) in the third year, 88% (22/25) in the fourth year and 96.4% (27/28) in the fifth year students in pediatrics (“5 + 3”), and the total response rate was 88.3% (68/77). The number of fifth-year students majoring in clinical medicine (“5 + 3”) and clinical medicine (5-year) was 36 and 20, respectively. Thus, a total of 124 students participated in the questionnaire. Among the participants, approximately 46% were male and 54% were female.

### Perception regarding scientific research among the students majoring in pediatrics (“5 + 3”)

**Fig. 1:**
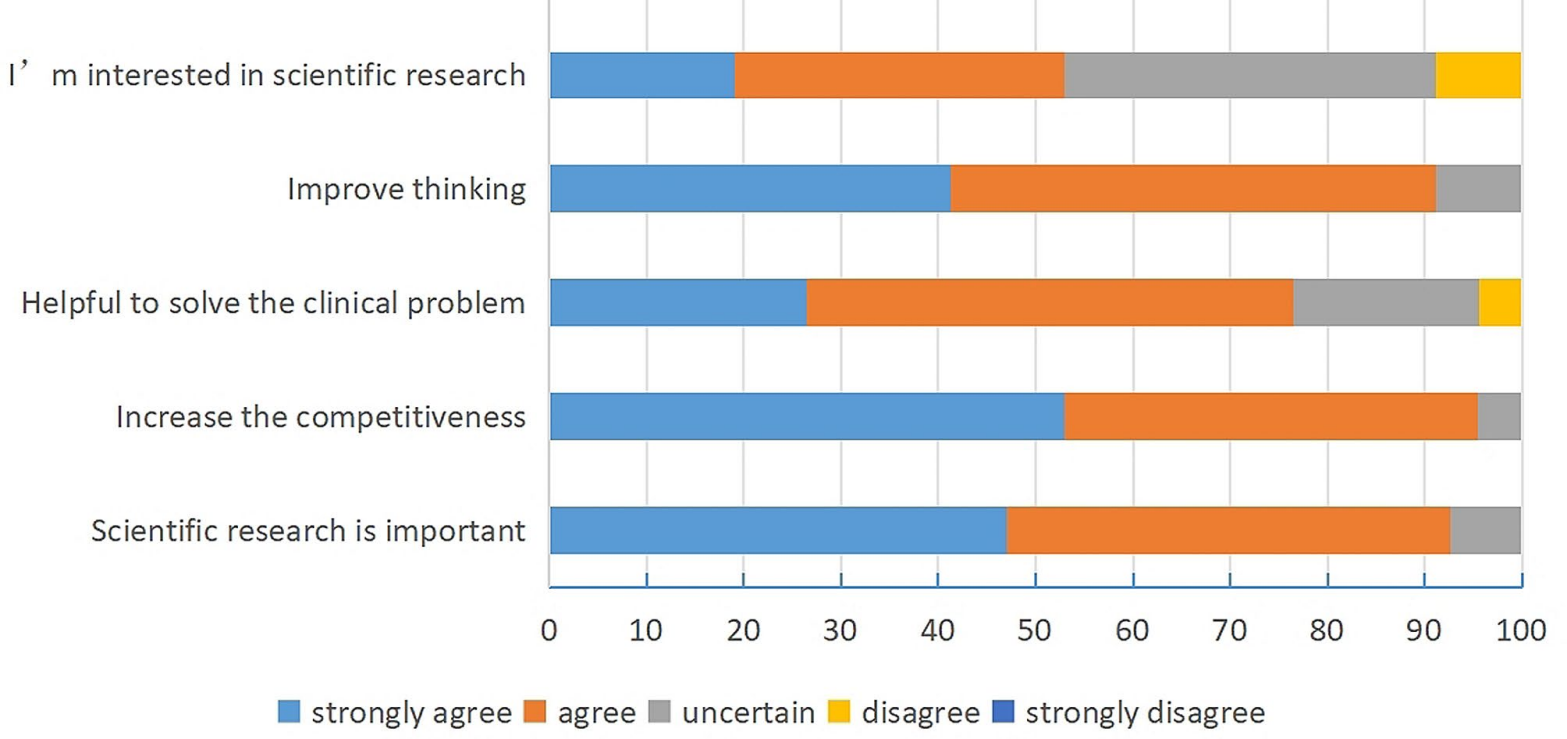
Perception regarding scientific research among the students majoring in pediatrics

The majority of students in pediatrics (“5 + 3”) recognized that research was important (92.6%), such as increasing competitiveness, solving clinical problems and improving thinking (Fig. 1). Approximately half of the students in pediatrics (“5 + 3”) were interested in the research.

Among the third-, fourth-, and fifth-year students in pediatrics (“5 + 3”), there was a significant difference in the effect of research on thinking ability (Table 1). A stronger understanding of the importance of research for thinking abilities was found in students from the fifth year.

**Table 1:** Comparison of perceptions of medical research based on different grades in pediatrics (“5 + 3”)

Pediatric (“5 + 3”)	Third-year (*n* = 19)	Fourth-year (*n* = 22)	Fifth-year (*n* = 27)	*P* value
Scientific research is important	4.26 ± 0.45	4.40 ± 0.67	4.48 ± 0.70	0.281
Increase the competitiveness	4.26 ± 0.56	4.5 ± 0.60	4.63 ± 0.56	0.111
Helpful to solve the clinical problem	3.95 ± 0.62	3.82 ± 0.96	4.15 ± 0.77	0.429
Improve thinking	4.26 ± 0.56	4.09 ± 0.68	4.56 ± 0.58	**0.031**
I’m interested in scientific research	3.47 ± 0.90	3.68 ± 0.95	3.70 ± 0.87	0.666

The bold values in the table denote *P* values less than 0.05 (indicating a significant difference)

Comparing the perception of medical research among the fifth-year students from the different medicine programs, there was a significant difference in the interest in research (Table 2). The fifth-year undergraduates from clinical medicine (5-year) received the highest score for interest in scientific research, followed by pediatrics (“5 + 3”).

**Table 2:** Comparison of perceptions of medical research among fifth-year students from different medicine programs

		Pediatric (“5 + 3”)	Clinical medicine (“5 + 3”)	Clinical medicine (5-year)	*P* value
Scientific research is important	agree (%)	88.9	88.9	95	0.700
	Uncertain (%)	11.1	5.5	5	
	disagree (%)	0	5.6	0	
	score	4.48	4.33	4.5	
Increase the competitiveness	agree (%)	96.3	100	100	0.673
	Uncertain (%)	3.7	0	0	
	disagree (%)	0	0	0	
	score	4.63	4.64	4.75	
Helpful to solve the clinical problem	agree (%)	77.8	63.9	70	0.197
	Uncertain (%)	22.2	27.8	30	
	disagree (%)	0	8.3	0	
	score	4.15	3.81	4.2	
Improve thinking	agree (%)	96.3	91.7	100	0.105
	Uncertain (%)	3.7	8.3	0	
	disagree (%)	0	0	0	
	score	4.56	4.36	4.7	
I’m interested in scientific research	agree (%)	59.3	47.2	85	**0.025**
	Uncertain (%)	33.3	36.1	10	
	disagree (%)	7.4	16.7	5	
	score	3.7	3.44	4.2	
	Uncertain (%)	7.4	11.1	10	
	disagree (%)	0	5.6	10	
	score	4.26	4.28	4.05	

The bold values in the table denote *P* values less than 0.05 (indicating a significant difference)

### Practices regarding scientific research among students majoring in pediatrics (“5 + 3”)

More than half of the students in pediatrics (“5 + 3”) participated in research training. Approximately 36.8% of them were involved in writing scientific articles, and 35.3% participated in research projects (Table 3). Only 4.4% of the students in pediatrics (“5 + 3”) contributed to publishing a scientific article, and 14.7% of the students in pediatrics (“5 + 3”) had attended medical congresses. However, none of the students had made a presentation at congresses.

**Table 3:** Comparison of the frequency of participation in research work in the different grades of Pediatric (“5 + 3”)

Pediatrics (“5 + 3”)	Third-year (*n* = 19)	Fourth-year (*n* = 22)	Fifth-year (*n* = 27)	*P* value
Project	7(36.8%)	8(36.4%)	9(33.3%)	0.963
Training	8(42.1%)	15(68.2%)	12(44.4%)	0.160
Write paper	8(42.1%)	6(27.3%)	11(40.7)	0.530
Publish paper	0(0)	1(4.5%)	2(7.4%)	0.775
As the first author	0	1	1	
Attend academic conference	0(0)	1(4.5%)	9(33.3%)	**0.002**
Conference communication	0	0	0	

The bold values in the table denote *P* values less than 0.05 (indicating a significant difference)

A statistically significant difference was observed among different grades in the pediatrics (“5 + 3”) program, with fifth-year students having a much higher rate of participation in conferences. However, no significant differences were observed in other forms of medical research practices.

When compared with fifth-year students from other programs (clinical medicine “5 + 3” or 5-year), the students in pediatrics (“5 + 3”) had a lower rate of participation in the projects (Table 4). The rate of participation in the research training of the pediatric students was lower than that of clinical medicine (5-year) (44.44% vs. 75%). There were no significant differences in other research practices, such as writing articles and attending congress.

**Table 4:** Comparison of the frequency of participation in research work among fifth-year students from different medicine programs

	Pediatric (“5 + 3”) (*n* = 27)	Clinical medicine (“5 + 3”) (*n* = 36)	*P* value	Pediatric (“5 + 3”) (*n* = 27)	Clinical medicine (5-year) (*n* = 20)	*P* value
Project	33.3%	66.7%	**0.009**	33.3%	75%	**0.005**
Training	44.4%	58.3%	0.275	44.4%	75%	**0.036**
Write paper	40.7%	50%	0.466	40.7%	50%	0.528
Publish paper	7.4%	16.7%	0.275	7.4%	5%	0.739
As the first author	3.7%	5.6%	0.733	3.7%	5%	0.828
Attend academic conference	33.3%	38.9%	0.650	33.3%	15%	0.154
Conference communication	0	0	NA	0	0	NA

The bold values in the table denote *P* values less than 0.05 (indicating a significant difference)

### Barriers regarding scientific research among the students majoring in pediatrics (“5 + 3”)

The most common barriers to research work for pediatric students were lack of training (85.3%), lack of time (83.9%), and lack of mentorship (82.4%).

However, the top three barriers to research work in fifth-year pediatric students were lack of training (96.3%), limited English (88.89%) and lack of time (88.89%). We found that the barrier of “lack of training” became increasingly apparent with grade, which was significantly obvious in fifth-year pediatric students compared with other grades (Table 5). The other barriers had no significant differences among the three grades from the pediatrics (“5 + 3”) program.

**Table 5:** Barriers toward research work in the different grades of Pediatric (“5 + 3”)

	Third-year (*n* = 19)	Fourth-year (*n* = 22)	Fifth-year (*n* = 27)	*P* value
Lack of time	78.9%	90.9%	81.5%	0.501
Lack of money	36.8%	68.2%	59.3%	0.115
Lack of laboratory	42.1%	59.1%	63.0%	0.379
Lack of mentorship	68.4%	86.4%	88.9%	0.223
Lack of training	63.2%	90.9%	96.3%	**0.007**
English is limited	57.9%	77.3%	88.9%	0.055
Lack of college attention	21.1%	31.8%	48.1%	0.156

The bold values in the table denote *P* values less than 0.05 (indicating a significant difference)

When compared with fifth-year students from other programs (clinical medicine “5 + 3” or 5-year), the rate of agreement about the barrier of “limited English” was significantly higher in fifth-year students from the pediatrics (“5 + 3”) program. There were no significant differences in other barriers among fifth-year students from different majors (Table 6).

**Table 6:** Comparison of the barriers to medical research among fifth-year students from different medicine programs

	Pediatric (“5 + 3”) (*n* = 27)	Clinical medicine (“5 + 3”) (*n* = 36)	*P* value	Pediatric (“5 + 3”) (*n* = 27)	Clinical medicine (5-year) (*n* = 20)	*P* value
Lack of time	81.5%	88.9%	0.406	81.5%	90%	0.417
Lack of money	59.3%	66.7%	0.546	59.3%	70%	0.449
Lack of laboratory	63%	75%	0.303	63%	70%	0.615
Lack of mentorship	88.9%	88.9%	1	88.9%	80%	0.397
Lack of training	96.3%	86.1%	0.173	96.3%	85%	0.170
English is limited	88.9%	55.6%	**0.004**	88.9%	65%	**0.048**
Lack of college attention	48.1%	41.7%	0.608	48.1%	40%	0.579

The bold values in the table denote *P* values less than 0.05 (indicating a significant difference)

### The type of research activities willing to involve in the future among the students majoring in pediatrics (“5 + 3”)

**Fig. 2:**
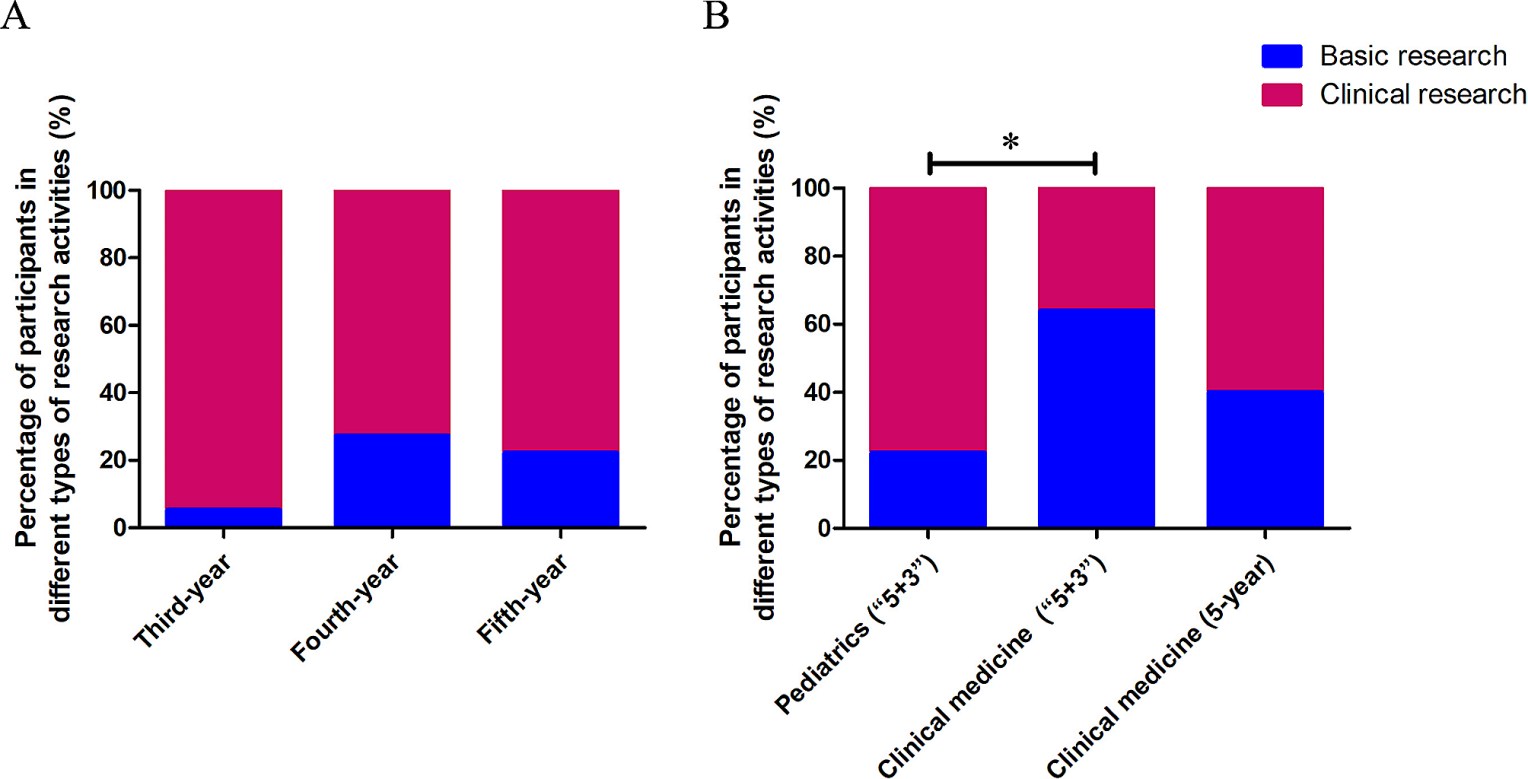
Types of research activities that students majoring in pediatrics are willing to be involved with in the future (**A**). Types of research activities that the students from different programs are willing to be involved with in the future (**B**). When compared with students in clinical medicine (“5 + 3”), fifth-year students in pediatrics (“5 + 3”) were significantly less likely to participate in basic research (*P = 0.001)

A total of 88.2% of students in pediatrics (“5 + 3”) wanted to participate in the training of scientific research activities. Furthermore, when asked about the type of future scientific research activities, 80.9% of students wanted to participate in clinical research, and only 19.1% of students wanted to be involved in basic research. There was no significant difference in the different grades of the students from the pediatrics (“5 + 3”) program (Fig. 2A).

Compared with students in clinical medicine (“5 + 3”), fifth-year students in pediatrics (“5 + 3”) were significantly less likely to participate in basic research (Fig. 2B).

## Discussion

In China, to solve the shortage of pediatricians, pediatric programs have resumed in some medical schools, including Zhejiang University, in recent years. In this study, we focused on the perceptions, practices and barriers to scientific research in pediatric undergraduates from Zhejiang University.

With global progress, more research is required to advance knowledge and innovation in all fields. Likewise, at the present time, research activities are a highly important skill for medical practitioner. Medical students were encouraged to take active part in scientific research and prepare for today’s knowledge-driven world [2]. In the current study, we found an overall positive perception of scientific research in pediatric undergraduates. More than 90% of pediatric students agreed (“strongly agree” and “agree”) that scientific research was important, which could make them more competitive and improve their thinking.

Although the students had a positive perception of medical research, their practice of conducting research remained unsatisfactory. When compared with the fifth-year undergraduates from clinical medicine (“5 + 3”) (66.67%) and clinical medicine (5-year) (75%), only 33.33% of the fifth-year undergraduates in pediatrics (“5 + 3”) have participated in scientific research projects. The number of paper publications was very small (third-year of Pediatric (“5 + 3”) 0, fourth-year 4.5% and fifth-year 7.4%). It was significantly less than the publication rate of final-year students in the United States (46.5%) and Australia (roughly one-third) [10, 11]. In another study in Romania, 31% of fifth-year students declared that they had prepared a scientific presentation for a medical congress at least once [12]. Moreover, none of the students in the study presented their paper in the scientific forum. A study in India also found that the undergraduate students’ experience of presenting paper in scientific forums was only 5% and publication 5.6% [13]. As part of the curriculum, some Indian universities require postgraduates to present papers and submit manuscripts for publication. Nevertheless, the practices regarding scientific research of undergraduates is still relatively poor. Lack of time, lack of guidance and lack of training for research careers were found to be the major obstacles in medical research for both pediatric students and others, which is consistent with previous reports [5, 14, 15]. The questionnaire in residents also found that lack of time was a critical problem for scientific research [16]. There is no common practice about how to solve this difficulty. In the literature, it was usually recommended that integration of scientific research training into the curricular requirements for undergraduates or residency programs for residents should be implemented [7, 14, 17, 18]. An increasing number of medical schools have individual projects as a component of their curriculum or mandatory medical research projects to develop research competencies [19, 20].

Interestingly, in fifth-year pediatric undergraduates (“5 + 3”), English limitations were found to be one of the most common barriers. The barrier of the limitation of English was increasingly better as the grades increased in pediatric students. We speculated that this was related to the increasing awareness of the importance of scientific research and participation in scientific research activities, increasing demand for reading English literature and writing English articles. Furthermore, the English limitation barrier for pediatric students was more obvious than that for students from clinical medicine (“5 + 3”) and clinical medicine (5-year). They are worried about academic English. Horwitz et al. first proposed “foreign language anxiety” [21]. Deng and Zhou explored medical students’ medical English anxiety in Sichuan, China. They found that 85.2% of the students surveyed suffered moderate above medical English anxiety [22]. In the questionnaire, 88.89% of the fifth-year pediatric students believed that limited English was one of the most important barriers for scientific research. Currently, English is the chief language of communication in the field of medical science, including correspondence, conferences, writing scientific articles, and reading literature. Ma Y noted that medical English should be the most important component of college English teaching for medical students [23]. At Zhejiang University, all of the students, including those majoring in pediatrics (“5 + 3”), clinical medicine (“5 + 3”) and clinical medicine (5-year), had a medical English course during the undergraduate period. Thus, the course could not satisfy the demands for scientific research, such as reading English literature, writing English paper and oral presentation in English. To solve this barrier, it was suggested to understand the requirements of pediatric students for medical English learning and offer more courses about medical English or English writing training for pediatric students. Furthermore, undergraduates should be encouraged to participate in local, regional or national conferences that are not in English but in Chinese language, which can increase the interest in participating in scientific research.

Most of the pediatric students tended to choose clinical research, while only 19.1% wanted to attend basic research. The proportion of fifth-year students in pediatrics (“5 + 3”) choosing basic research was much lower than the students from the clinical medicine (“5 + 3”) program. It is speculated that pediatrics usually have heavier clinical work with relative poor scientific practice in China, compare with doctors from other clinical department. They are likely to concern the clinical research. The students in pediatrics might not obtain sufficient scientific guidance from their clinician teachers compared with those from other medicine program. According to the data, the Pediatric College could conduct more scientific research training directed at clinical research, such as the design, conduct and administration of clinical trials. The simulation-based clinical research curriculum is considered to be a better approach training of clinician-scientists compared with traditional clinical research teaching [24]. On the other hand, we might need to do more to improve the interest in basic research for pediatric undergraduates.

The major limitation of the present study is the small sample size. Only 20 to 30 students have been enrolled in pediatrics (“5 + 3”) of ZUSM every year. Therefore, multicenter studies (multiple medical schools) might be better to understand the perception, practice, and barriers of medical research among pediatric undergraduates. Even so, the findings in this study indicate that lack of time, lack of guidance, lack of training and limited English might be the common barriers to scientific work for pediatric undergraduates. Furthermore, the questionnaire for teachers and administrators would be performed to offer some concrete solutions in future.

## Conclusions

Although pediatric undergraduates recognized the importance of medical research, interest and practices in research still require improvement. Lack of time, lack of guidance, lack of training and limited English were the common barriers to scientific work. Therefore, research training and English improvement were recommended for pediatric undergraduates.

## Data Availability

The datasets used and/or analyzed during the current study are available from the corresponding author upon reasonable request.

## Abbreviations

ZUSM: Zhejiang University School of Medicine
KMO: Kaiser-Meyer-Olkin
